# Academy of Stomatology

**Published:** 1903-12

**Authors:** 

**Affiliations:** Academy of Stomatology


### ACADEMY OF STOMATOLOGY.

A STATED meeting of the Academy of Stomatology of Philadelphia was held at its rooms, 1731 Chestnut Street, on the evening of Tuesday, March 24, 1903.

Papers were read by Dr. D. R. Stubblefield, of Nashville, Tenn., entitled "We made Better Dentists then than now," and Dr. S. M. Weeks, of Philadelphia, entitled "The Importance of the Mesio-Distal Relation of the Two Arches during Childhood."

(For Dr. Stubblefield's paper, see page 917; for Dr. Weeks's paper, see page 842.)

### DISCUSSION OF DR. STUBBLEFIELD'S PAPER.

*Dr. E. C. Kirk.*—In attempting to open the discussion of this question, I fear that I have been set a difficult task. It has not been stated in the paper just who are meant by "they," nor has the essayist stated whom he means by "we." These are two very important factors. The "they" I have any knowledge of from personal experience do not date back more than twenty-five years. Back of that knowledge of educational conditions has been obtained from the literature of our profession. I do not believe that the gentlemen who uttered the statement quoted as the title of the essay meant what that title would be taken to mean read as a bald statement. It must need qualification from the fact that the essayist says it was a statement made by one of the best men in dentistry. It seems impossible that in our growth of knowledge of detail which makes up our educational system we have taken a backward step. If the statement means that we actually did make better dentists then than now, I frankly state that I do not believe it; because within my recollection it was quite customary to talk about such things as "laudable pus." We know now that pus is not laudable. We knew very little about the conservative treatment of the dental pulp. We have gone the whole gamut, and are beginning to view the operation of pulp-capping somewhat askance. We knew little about the etiology of dental caries, though men would attempt to explain the tendency of teeth to decay on the proximal surface upon the theory of lateral pressure. It may seem insignificant that a wrong conception of theory should have any particular bearing on our work, but as an outgrowth of that conception we had methods for the absolute mutilation of the approximal surfaces, because men believed in the production of caries by proximal contact. It took some years of scientific education to get rid of that heresy, and upon this newer education we have based a rational mode of practice. The men who know these things are better dentists than those of years ago who did not know them. In regard to manipulative skill, they had some wonderful operators then. (I am speaking of the "they" within my recollection.) I was talking with a man from Kansas City on this topic, and he said that twenty-five years ago he was the only dentist in Kansas City capable of putting in a decent contour cohesive gold filling. To-day there is scarcely a village throughout this land where we will not find a man capable of doing this work. I think that twenty-five years ago there were more men especially called to practise dentistry in the sense that they had natural ability and enthusiasm for their work. I read recently in an old copy of the *New York Dental Recorder* an advertisement of the old Baltimore College of Dental Surgery. The winter courses were from November to February, and the men attending the medical course were permitted to come up for the degree at the end of one winter's course. Dr. Stubblefield said they assimilated the courses better then than now. Naturally, for they did not have much to assimilate. The data of dentistry had not grown then to present proportions. The men who make this camparison to the detriment of modern dental conditions seem to overlook the fact that the data has accumulated to such an extent that it is beyond the ability of the ordinary mind to grasp it at once. This was illustrated at an Alumni meeting at Buffalo when an instructor was making a plea for laboratory instruction in medicine. Later in the evening an older alumnus said, "I do not want to cast any reflections upon this modern method of teaching, but I belong to that class who graduated some thirty or forty years ago, and I ask, What has become of the old-fashioned physician? We have nothing but specialists now. The old practitioner is occasionally making use of these laboratory experts. They send material for a report to be used as a means of diagnosis. In the course of time a paper is sent back with a lot of figures and new words and a lot of data extremely interesting, but what in the devil does it all mean?" That is the point. It is the business of the modern practitioner to know what it all means, and it is the business of the school to teach what it all means.

The part of the paper in which the essayist deals with the matter of education I think presents the most important phase of the question. He gave us a very interesting illustration of his own experience in finding that a quiz-master was a better teacher than one of the learned professors. I think dentists have gone astray on the question of terms. We hear "hard" and "soft" gold spoken of when what is meant is cohesive and non-cohesive gold. Another vagary of the dental man is that the theoretical man and the practical man represent two distinct branches. A theory is one of the most practical things in the world. A man may know how, and not be able to do a thing; but a man cannot do it unless he knows how. Do not let us make part of our creed the heresy that this scientific or physiological training is useless. A man cannot, in my judgment, properly prepare a cavity for a gold filling unless he knows something about bacteriology.

I am glad that Dr. Stubblefield called attention to the question of the fourth year. I hope the time of the fourth year will be so utilized that, instead of adding new data to the curriculum, time will be afforded for teaching thoroughly what we now attempt to teach in three years.

*Dr. James Truman.*—I suppose it is necessary for us all to have something to say on this subject, especially we of the older class. It is a habit, I believe, with all the older men to say that the former days were better than these. I do not belong to that class, and yet while I say that I want to be understood. The older days in some respects were better than these, and the present is infinitely better in other respects than the older days. We must make a distinction. When we go back to the very beginning of the dental profession, so to speak,—I mean the origin and formation of college life,—those of us who had any experience in that direction knew well how limited was the instruction. The man who wanted to study anatomy, for instance, received but a modicum of the science. Chemistry was not, physiology was not, and, may I say it? mechanical dentistry was not. This was because the men who came into dentistry along in the fifties were men who studied under other dentists, who received their mechanical education under the fathers of the profession, and coming into college they cared nothing for the mechanical and a great deal for the operative instruction. The mechanical part was neglected, and up to a period in the seventies there was practically no mechanical dentistry taught at any of the colleges that I was acquainted with. Then came a change, and if there has been any change that has been to the advantage of the dental profession, it has been that the technique of mechanical dentistry has been more thoroughly elucidated during the last twenty-five years. In my opinion, in the proportion as that branch has advanced, operative technique has lost ground. This is probably because we have undertaken to do too much in this present day. I am in sympathy with the broader education of the present time, but I am sure that the men do not digest what they receive. There is but a partial education all the way through. I do not agree with my colleague that it is necessary for a man to understand bacteriology in order to prepare a cavity correctly. Much as I admire bacteriology, I feel that the doctrine of "extension for prevention" is the throwing of sand into the eyes of the operator of the present day. I believe in thorough education. I believe that if you want a man to know anything at all you must drill it into him, and that cannot be done in a few days or a few weeks. He must have time to digest that which he has taken into his mental organism. We have not had sufficient time for that, and this is the reason why we are not making better practical dentists to-day than were made when I was young in dentistry.

The operative branch to-day is not equal to what it was forty years ago. When I say that I mean that we knew how to fill teeth fifty years ago. I said that at the last meeting of this Association when Dr. Rhein talked about the improvement that had come about since Dr. Webb had come into this profession. We did not have all the appliances that we have at the present time, and it required more skill with the few that we had at that period. The past was the foundation upon which all the present is built. We must endeavor to arrange the curriculum that the individual will take that which seems best adapted to his work and endeavor to do it thoroughly. I do not want to be misunderstood here. I am just as much in favor of that broader education as my friend Dr. Stubblefield, or as Dr. Kirk, but I want every individual to be as thorough as possible in what he acquires.

It has been said that men were older when they came into the profession a few years ago. That is true. They were older because they had been with their preceptors. We find to-day a better educated class of men entering the profession, but they enter without any idea of their fitness to become dentists. I regret we have such a class of students to deal with. Twenty-five or more years ago men knew why they wanted to become dentists; now it is more often what the parents or guardians desire.

*Dr. S. H. Guilford.*—It is very difficult to decide a question of this kind on account of the many problems presenting. There is something to be said on all sides. As has been said, men who studied dentistry twenty years ago were men who had some decided taste for this sort of work. This was shown in the boy who would take clocks apart or meddle with locks or water-wheels. In my own case I was led into dentistry simply because I was constantly tinkering at something. At that time, too, the boy entering dentistry was put on a system of probation to ascertain whether he was fitted for dental work. If he was not, he was dropped. To-day young men enter dentistry for the reason that they think they can make more money and make it more easily than in some other way. Looking at the matter in that light, I believe more good dentists were turned out forty years ago than to-day.

In regard to the making of dentists, they did not make the dentists; the dentists made themselves. When I went to college we had a good course in anatomy, but of the other branches we did not have very much that we could be proud of. We secured, however, a little foundation upon which we could build. If I know anything of dentistry to-day, I have learned it nearly all since I left college. It is not necessary that our students assimilate all of the curriculum; if we take them over the curriculum and get them into the position where they can learn after leaving college we are doing well with them. A man must have ability and knowledge, and if he has the ability he will work, and there is no reason why he should not be a success. I wish there were some way to-day of finding out when a student enters college whether he has natural qualifications for the practice of dentistry. Any profession is better when made up of men especially fitted for the work. I do not think the number of branches in the curriculum needs to be increased, but more thorough drilling in the present branches is needed.

*Dr. E. T. Darby.*—I was present in Chicago at the Institute of Pedagogics, and heard the statement made which has been the text of the evening. It was made when the subject for discussion was "The Fourth Year in Dental Schools." I also heard the gentleman who made the statement modify it at the close of his remarks by saying that we made better practical dentists twenty years ago than we make to-day. The statement was made with the idea of emphasizing the importance of giving the student in the fourth year a great deal of practical work. I heard this same man say at that time that no one was more in favor of broad culture, of thoroughness in training, than himself, and that he believed in giving the student everything that it was possible to give him in the way of culture commensurate with making a good practical dentist of him. I do not believe that that man believed in giving the student less, but in giving him more, practical work. It is by doing over and over and over again those things that we do with our fingers that we become dexterous in their doing. We have crowded into our three years so much that is valuable, so much that students ought to know, that we have sacrificed to a large extent the time that ought to be devoted to practice. For that reason I believe that that statement was true, that when twenty years ago we had very much less in our curriculum than we have now, and expected very much less of our students than we do to-day, and when they devoted so much more time to work in the laboratory or in the clinic-room, they were better practical men than the men turned out to-day.

For this reason I shall hail with delight the addition of the fourth year to our course. I would not take anything from our present college course, but I would make the men good practical dentists before we send them out. If the men who have a fitness for dentistry would enter our schools, we would have no difficulty in turning out good dentists.

*Dr. Kirk.*—I would like to ask Dr. Darby if, in his judgment, it is not true that if we do give sufficient time to the development of manipulative skill in the student, with this increased culture we will make better dentists to-day than we did then?

*Dr. Darby.*—We will make better dentists with the four years'

course simply because we will give the students more practice in addition to the better scientific foundation.

*Dr. Jesse Green,* West Chester, Pa.—I did not come to this meeting to make a speech, but to hear what others had to say. These men who have gone back twenty-five or thirty years seem to me to be boys. That which the essayist has said has struck me forcibly. I come before you as one who does not know anything. When I was a boy we read a few books, but we studied them thoroughly. To-day we read so much that it is impossible to retain it for any length of time. When I was a boy the one who had some talent for mechanical pursuits and tinkering was looked upon as one who would make a good mechanic. I studied with a man who was a mechanical dentist. When I asked him about some of the tissues of the body he did not know anything about them. It is often the case with men who know how to do a thing that they cannot tell why they do it. It is like the man who has charge of our town clock. "Well," he says, "it is so, and you know it is so, and that is enough." That was the way we were. We read a great deal of what had been said about the pulp, but we knew little about it. We were working gradually and building practical knowledge upon practical knowledge. We were trying to make ourselves dentists. The word "dentist," we thought, covered everything. I think the part in our teaching to be improved upon to-day is the practical work. While there has been great progress in dentistry, when it comes to mechanical work, up to the time of bridge-work, I think some of the prosthetic work done fifty years ago was probably equal to some of the mechanical work, outside of bridge-work, that we see to-day.

*Dr. R. H. Nones.*—As has been said, the men who entered dentistry twenty-five years ago were selected men. They received their first instruction under a preceptor, and, fortunately or unfortunately for them, they were told before entering college whether they were fitted for the study. In making comparisons with students of other days we forget that those men have grown in the profession. We might ask the question, What will the present practitioner be in the future? In twenty-five years I venture to say there will be no comparison between the work of men who will have been in actual practice twenty-five years and those men who to-day have had twenty-five years of experience. Twenty-five years ago a man who had been in actual practice five years could graduate in one year. I agree with Dr. Kirk that, given the extra amount of education with the same amount of manipulative ability, our graduates to-day cannot help being better dentists than were made twenty-five years ago. An unfortunate fact connected with the candidates for entrance that we receive to-day is the lack of manipulative ability. The requirements for entrance state that the candidate must have been two years in a high school. Twenty-five years ago every student who graduated was considered competent to practise; to-day he is obliged to confront an examining board.

My idea in regard to the fourth year is that if it be regarded as a post-graduate course, and used for practical instruction, it will be exactly what is needed.

*Dr. M. H. Cryer.*—I believe that to-day I am a better dentist than yesterday, and that this year we will turn out better dentists than we did last year, and that last year we turned out better men than we did the year before. We treat teeth better than we did formerly. We take better care of the mouth and the system than we ever did, and I believe that we are advancing all the time. It is not the time to look at what is past. I believe that as we advance and add to our knowledge the things that are necessary we will make better dentists than we did in the past.

*Dr. William Trueman.*—We must all recognize the fact that systematic dental education has not yet passed the experimental stage. This is impressed upon us by the fact that the last survivor of those starting our dental college is only eighty-three years of age. The man who qualified himself for his life work by laboring from ten to twelve hours a day for years was better prepared than some from our colleges to-day. He learned how to make metal plates fit to eat with. The question is, How shall we make better dentists? We do not want to go back to the times when nothing was known of bacteriology. To-day the student leaves college knowing how things should be done, but has not that knowledge at his fingers' ends. The addition of this manipulative training to scientific education is what is needed.

*Dr. Stubblefield* (closing.)—I feel the same embarrassment about closing my paper that I had in presenting it. I am not so recreant to modern progressive ideas as to believe that we have not improved upon the past. No college can make a man, but the college can lay a foundation upon which that man can intelligently make the superstructure. In regard to the fourth year, my own conviction has been from the first that it was to be an opportunity to readjust, if necessary, the teaching; certainly to put the student and teacher in such relation as to get the best out of the possibilities; to stimulate the students to higher ideals and better practical results.

I congratulate myself that I did agitate your minds, which was the best I could hope to do, and I thank you very much for the attention you have given me.

DISCUSSION OF DR. WEEKS'S PAPER.

*Dr. G. L. S. Jameson.*—I have enjoyed this paper very much. I know that the doctor has given more thought to the subject than the majority of practitioners. I congratulate him upon his results, and I will say that he has been very helpful to me in certain lines of malocclusion. I think he is correct in saying that we should begin very early to look out for malocclusions and irregularities, and to do what we can from the very first to correct them. I try to see my own cases when they are from four to eight years old. There is little to be done but to see that the first teeth are extracted at the proper time and to look out for the first permanent molars, which should be given attention from the very first.

*Dr. H. B. Hickman.*—I am sorry that the men who discussed the first paper are not here to discuss this one, because this is new dentistry and not dentistry of twenty-five years ago. The men graduating to-day are better qualified than those graduating five years ago. When I graduated little was known of orthodontia. If a man got a case, he got rid of it as soon as possible. I have devoted a good deal of time to the subject of orthodontia, and have been greatly helped by the assistance of Dr. Weeks. I feel under obligation, first, to Dr. Angle, and second, to Dr. Weeks.

*Dr. Weeks* (closing).—I feel that the closing of the discussion is a matter of very little moment. I am surprised that I have not been brought to account for some things said in my paper. I thank you for your attention.

OTTO E. INGLIS,
*Editor Academy of Stomatology.*

